# Effect of microalgal biostimulants (*Chlorella vulgaris* and *Arthrospira platensis*) on the growth performance of Pak Choy in a hydroponic system

**DOI:** 10.21315/tlsr2026.37.3.5

**Published:** 2026-10-15

**Authors:** Isha Rasheed, Fong-Lee Ng, Ming-Li Teoh, Nallammai Singaram, Georgepeter Gnana kumar, Fiona Seh-Lin Keng, Sze-Looi Song

**Affiliations:** 1 School of Biosciences, Faculty of Health and Medical Sciences, Taylor’s University, 47500 Subang Jaya, Selangor, Malaysia; 2 Food Security and Nutrition Impact Lab, Taylor’s University, 47500 Subang Jaya, Selangor, Malaysia; 3 Centre for Active Living, Taylor’s University, 47500 Subang Jaya, Selangor, Malaysia; 4 Department of Physical Chemistry, School of Chemistry, Madurai Kamaraj University, Madurai-625021, Tamil Nadu, India; 5 Institute of Biological Sciences (ISB), Faculty of Science, Universiti Malaya, 50603 Kuala Lumpur, Malaysia; 6 Institute of Ocean and Earth Sciences, Universiti Malaya, 50603 Kuala Lumpur, Malaysia; 7 Institute for Advanced Studies, Universiti Malaya, 50603 Kuala Lumpur, Malaysia

**Keywords:** Hydroponic System, Biostimulants, Sustainable Agriculture, *Chlorella vulgaris*, *Arthrospira platensis*, Sistem Hidroponik, Biostimulan, Pertanian Lestari, Chlorella vulgaris, Arthrospira platensis

## Abstract

The growing interest in sustainable agricultural practices has led to an exploration of biostimulants. This study investigates the impact of microalgae as biostimulants in a hydroponic system, using Pak Choy as the model plant. Three hydroponic setups were employed: a control system containing tap water with hydroponic fertiliser and two experimental systems supplemented with microalgae without fertiliser— *Arthrospira platensis* and *Chlorella vulgaris*, respectively. The control group produced the tallest plants and the highest fresh weight, exceeding the other treatments by 106.65 mm in height and 9.344 g in fresh weight. Between the two microalgal treatments, *C. vulgaris* produced higher plant height, fresh weight and improved physicochemical indicators compared to *A. platensis*. While both microalgal treatments yielded lower biomass than the fertilised control, they were able to sustain plant growth in the absence of synthetic fertiliser.

## Abstrak

Peningkatan minat terhadap amalan pertanian lestari telah membawa kepada penerokaan biostimulan. Kajian ini menyiasat kesan mikroalga sebagai biostimulan dalam sistem hidroponik, menggunakan Pak Choy sebagai model tanaman. Tiga persediaan hidroponik telah digunakan: sistem kawalan yang mengandungi air paip dengan baja hidroponik dan dua sistem eksperimen yang ditambah dengan mikroalga tanpa baja—Arthrospira platensis dan Chlorella vulgaris. Kumpulan kawalan menghasilkan tanaman yang paling tinggi dan berat segar tertinggi, melebihi rawatan lain sebanyak 106.65 mm dalam ketinggian dan 9.344 g dalam berat segar. Antara dua rawatan mikroalga, Chlorella vulgaris menghasilkan ketinggian tanaman dan berat segar yang lebih tinggi serta meningkatkan indikator fizikokimia berbanding Arthrospira platensis. Walaupun kedua-dua rawatan mikroalga menghasilkan biomassa lebih rendah daripada kawalan yang dibaja, mereka mampu mengekalkan pertumbuhan tanaman tanpa baja sintetik.

**HIGHLIGHTS**

- *Chlorella vulgaris* and *Arthrospira platensis* sustained Pak Choy growth in hydroponics without synthetic fertiliser.
- *C. vulgaris* enhanced plant height, biomass and photosynthetic efficiency compared to *A. platensis*.
- Microalgal treatments influenced reservoir water quality and improved photosynthetic performance indicators in Pak Choy.

## INTRODUCTION

In the past century, the increasing challenges associated with global warming and urbanisation have significantly increased the awareness for sustainable and environmentally-friendly practices which extended to agriculture, an activity known to be resource intensive. Farming or agriculture has drastically changed from soil farming to hydroponic farming. Hydroponics is an approach to agriculture that uses nutrient-rich water instead of soil, offering various advantages over traditional farming methods (Dubey & Nain 2020; Rajaseger *et al*. 2023). The advantages of utilising a hydroponic system in agriculture include high yield quality, effective water and resource management, easy crop management and controlled spread of soil borne diseases (Kannan *et al*. 2022).

According to Gaikwad *et al*. (2024), conventional hydroponic systems are considered a promising agricultural approach, as they reduce plant diseases and enable crops to grow approximately 30%–40% faster compared to traditional cultivation methods. However, in most cases of poorly designed, conventional hydroponics systems, most nutrients in hydroponics media go wasted and are flushed, causing environmental degradation (Kumar & Cho 2014). Using biostimulants helps to enhance the nutrient uptake, stress tolerance and increase the production of quality crops. Microalgae and cyanobacteria are emerging biostimulants due to their renewable bioactive resources. These are photoautotrophic organisms with significant commercial value (Bello *et al*. 2021). Microalgae and cyanobacteria are becoming more and more recognised as beneficial biostimulants in sustainable agriculture due to their diverse biochemical profiles and multifunctional agronomic benefits over several conventional biostimulant alternatives. These microbes create a wide range of bioactive compounds, including polysaccharides, amino acids, vitamins, antioxidants and phytohormones (auxins, cytokinins, gibberellins). In both optimal and stressful conditions, these compounds can enhance root development, accelerate seed germination, increase chlorophyll and nutrient absorption, and improve overall plant performance. (Prisa & Spagnuolo 2023). Microalgae species like Chlorella and Spirulina produce auxins, cytokinins and gibberellins, which are plant growth regulators (Chiaiese *et al*. 2018). They promote processes like cell division, root elongation, lateral root formation and shoot growth, these substances have an impact on the hormonal balance of plants. The vitamins and mineral elements included in the microalgae along with polysaccharides, carotenoids and amino acids that enhance photosynthetic efficiency, stimulate chlorophyll synthesis and increase plant biomass and productivity (Chiaiese *et al. 2018*).

Two main microalgae used in this experiment are *Arthrospira platensis* and *Chlorella vulgaris*. *C. vulgaris* is known for improving photosynthetic pigment in plants (Suchithra *et al*. 2022). It is rich in chlorophyll a and b (Arora & Sahoo 2015), and contains essential amino acids, dietary fibres, minerals, proteins and antioxidants (Park *et al*. 2022). As a biostimulant, it has been reported to enhance shoot and root development in plants (Vildanova *et al*. 2023), although it is more commonly used as a biofertiliser (Suchithra *et al*. 2022). *A. platensis* is known for increasing the number of leaves and protecting the plant from heavy metal contaminants (Siringi *et al*. 2022). *A. platensis*, a filamentous cyanobacterium, rich in proteins and pigments including phycocyanin, allophycocyanin and chlorophyll a (Phang *et al*. 2000). Its biostimulant potential is linked to increased biomass under specific nutrient conditions and its ability to remove contaminants from wastewater (Chojnacka *et al*. 2005; Solisio *et al*. 2006; Pane *et al*. 2008). *C. vulgaris* contains essential elements such as nitrogen, magnesium and iron, which are important for the formation of chlorophyll molecules and photosynthetic proteins (Renuka *et al*. 2017). The presence of antioxidants that improve plant metabolism and protect the photosynthetic apparatus from oxidative stress. These effects enhance photosynthetic efficiency and overall plant growth. Studies have shown that application of *Chlorella* extracts significantly increases chlorophyll content and photosynthetic performance in treated plants (Chiaiese *et al*. 2018).

The primary objective of this study is to evaluate the biostimulatory effects of microalgae on the physiological performance and health of Pak Choy, as indicated by parameters such as plant height, biomass, leaf number, pigmentation and photosynthetic activity. *Brasica rapa* var. *chinensis (*Pak Choy) is being used as the model plant, as it is a highly versatile, fast-growing, short growth cycle well adapted to soilless systems (Laiza *et al*. 2022). In addition, Pak Choy thrives under regulated circumstances, producing consistent, high-quality harvests in a short period of time (McCandless 2023).

In addition, the study aims to assess the impact of microalgae application on the water quality of the hydroponic reservoir by monitoring indicators including dissolved oxygen (DO), electrolytic conductivity (EC), turbidity, pH, oxidation-reduction potential (ORP) and chemical oxygen demand (COD). The concentrations of orthophosphate (O-PO_4_^3−^), nitrate-nitrogen (NO_3_^−^-N) and ammoniacal-nitrogen (NH_3_-N) were also quantified to evaluate the influence of microalgal biostimulants on nutrient availability and uptake in the hydroponic system. This study provides preliminary insights into the potential role of microalgae as biostimulants in hydroponic systems, with possible implications for sustainable agriculture and improved crop quality in urban settings.

## MATERIALS AND METHODS

The experimental design is illustrated in Fig. 1. The study involved three main phases:

1. Cultivation of microalgae.
2. Germination of Pak Choy (*Brassica rapa* subsp. chinensis) seeds.
3. Evaluation of plant growth, microalgae characteristics and water quality in a hydroponic system.

### Preparation of Microalgae

Two microalgae species were used: *Chlorella vulgaris* (UMACC 038, Universiti Malaya Culture Collection) and *Arthrospira platensis* (obtained from AlgaeLiving, Malaysia). *A. platensis* was obtained from a commercial source, with purity verified by the supplier. Cultures were acclimated under laboratory conditions prior to use, and exponential growth was confirmed. Cultivation followed the method described by Phang and Chu (1999). *Chlorella vulgaris* was cultured in Bold’s Basal Medium (BBM), while *A. platensis* grew in Kosaric medium. Cultures were maintained under a 12:12 h light-dark cycle at 25 ± 2°C with 24 h aeration in an incubator (Protech, Incubators COOL-300) with light intensity 40 μmol m^−1^s^−1^ (Philips BN058C T5 White LED). Based on findings reported in our previous publications (Tay *et al*. 2024; Ng *et al*. 2021), the life cycle of microalgal cells is approximately 12 days. Accordingly, cultures were monitored daily by optical density measurements, and biomass was harvested at the exponential growth phase, which occurs at approximately day 5 of the growth cycle. Stock cultures were prepared by inoculating 10% (v/v) of exponential-phase cultures (OD_620_nm__ = 0.2) into fresh media. Media preparation followed Phang and Chu (1999).

### Germination of Pak Choy Seeds

F1 hybrid Pak Choy seeds (DPC88, Crop Power) were germinated in hydroponic sponges. Two blocks of 20 sponges were soaked in tap water, with air removed by compression. Each sponge received two seeds in its concave core. Two seeds were placed in each sponge to ensure at least one successful germination per sponge, thereby improving efficiency and minimising losses. The blocks were placed in a sunlit area for 7 days inside Taylor’s Urban Farm, an indoor facility with an enclosed environment equipped with a cooling system temperature range: 30 ± 5°C (see Fig. S1 in Appendix). Only uniform seedlings were selected for subsequent hydroponic experiments to ensure consistency across replicates.

### Hydroponic System Setup

Three independent hydroponic systems were established: a control, a *C. vulgaris* treatment, and an *A*. *platensis* treatment. Each system consisted of a two-tier recirculating setup with 10 planting sites and a 10 L reservoir. Each hydroponic system contained 10 plants, providing 10 biological replicates per treatment.

1. Control: 10 L of tap water with hydroponic fertiliser in a black tank to prevent light penetration and algal growth. Electrical conductivity was adjusted to ~2 mS/cm using hydroponic fertiliser (CityFarm, Malaysia).
2. *Chlorella vulgaris* treatment: 1 L of *Chlorella vulgaris* culture + 9 L of tap water in a transparent tank.
3. *Arthrospira platensis* treatment: 1 L of *A. platensis* culture + 9 L of tap water in a transparent tank.
4. Microalgae inoculum was standardised to 0.5 (OD_620_nm__) using a UV spectrophotometer (Secomam, France).

Uniformity across planting sites was maintained by using identical sponge blocks, seed placement, and nutrient solution preparation. Each hydroponic system was operated independently to ensure replication at the system level. Microalgal inoculum was standardised to OD_620_ = 0.5 using a UV spectrophotometer (Secomam, France). This value was selected based on preliminary growth curve data, where OD_620_ = 0.5 corresponded to mid-exponential phase, ensuring active and metabolically consistent cultures. Algal density was maintained over time by continuous mixing in the recirculating system, which minimised sedimentation and ensured even distribution of cells throughout the reservoir.

The experimental setup was located inside Taylor’s Urban Farm, an indoor facility with an enclosed environment equipped with a cooling system (temperature range: 30 ± 5°C) (see Fig. S2 in Appendix).

### Growth Measurements

Plant height and fresh weight were measured weekly for four weeks with ten plants per treatment serving as biological replicates. Plant height was defined as the distance from the base of the stem at the sponge surface to the tip of the longest leaf, measured using an electronic digital caliper (500-196-30 Mitutoyo, Japan). Leaf length was assessed by measuring the length with a digital caliper. Fresh weight was determined destructively at final harvest only, using a precision balance (AND EK-300i, Japan). For non-destructive measurements such as height and leaf area, plants were carefully removed from the hydroponic system to prevent root damage, measured and then returned to their respective systems. This standardised procedure was consistently applied across all treatments to ensure comparability and reproducibility.

### Photosynthetic Performance

Photosynthetic efficiency was assessed using an AquaPen-P fluorometer (AP 110-P, PSI, Czech Republic) following Ng *et al*. (2020). For each plant, one intact leaf was dark-adapted in situ by covering it with aluminum foil for 15 min prior to measurement; the leaf was not detached from the plant. Rapid Light Curves (RLCs) were generated using AquaPen software, with stepwise increases in light intensity applied to evaluate photosynthetic responses. Measurements were conducted under controlled indoor conditions inside Taylor’s Urban Farm to minimise environmental variability. The following parameters were obtained:

1. Fv/Fm: Maximum quantum yield of photosystem II, an indicator ofoverall photosynthetic health.
2. Alpha (α): Maximum photosynthetic efficiency, reflecting the initialslope of the RLC.
3. rETRmax: Relative electron transport rate at light saturation, representing the maximum photosynthetic capacity.
4. Ek: Photo-adaptive index, the light intensity at which photosynthesis transitions from efficiency-limited to capacity-limited.

These parameters were selected to provide insight into both plant physiological status and algal culture performance under experimental conditions. Three replicates were performed for each treatment setup to ensure reproducibility.

### Pigment Analysis

#### Chlorophyll-a (Chl-a)

The microalgae cells were then harvested by millipore filtration using filter paper (Whatman GF/C, 0.45 mm). Chl-a content was determined from 10 mL water samples filtered and extracted in 10 mL acetone. Samples were stored at 4°C for 24 h in the dark, then centrifuged. Absorbance was measured at 630 nm, 645 nm and 665 nm using a UV spectrophotometer (Secomam, France) (Ng *et al*. 2020; Thong *et al*. 2023). Chl-a concentration was calculated as:


$$\text{chl}-a\;(\text{mg }{\text{m}}^{-3})=(\text{CA}-\text{VA})/\text{VC}$$

where CA = 11.6 × OD_665_nm −1.31 × OD_645_ −0.14 × OD_630_, VA = volume of acetone (mL) used for chlorophyll extraction, and VC = volume of algal culture (L) and chl-a (mg L^−1^) = chl-a (mg m^−3^)/1000.

#### Phycobiliproteins (PBP)

The microalgae cells were then harvested by millipore filtration using filter paper (Whatman GF/C, 0.45 mm). Phycobiliproteins in *Arthrospira platensis* were extracted from 10 mL water samples filtered using 10 mL phosphate buffer (0.01 M of Dipotassioum Phosphate and 0.15 M of Sodium Chloride, pH adjust to 7). Absorbance was measured at 615 nm and 652 nm (Thong *et al*. 2023). The formulas to calculate phycocyanin (PC) and allophycocyanin (APC) were adopted from Stein-Taylor and America (1973).


$$\begin{array}{l} {}[\text{PC}]\;\text{mg }{\text{L}}^{-1}=({\text{OD}}_{615}-0.474\times {\text{OD}}_{652})/5.34\\ {}[\text{APC}]\;\text{mg }{\text{L}}^{-1}=({\text{OD}}_{652}-0.208\times {\text{OD}}_{615})/5.19 \end{array}$$

The concentrations of PC, APC or PE content in each volume of culture were determined using the following equation:


PC or APC (mg/L) in the culture cells=(C×Ve)/Vc

Where C = concentration pf PBP (PC or APC), Ve = volume of extract (mL) and Vc = volume of culture (mL).

### Water Quality Analysis

Water samples were collected from the reservoirs of each hydroponic setup throughout the experimental period. Samples were collected in sterile polyethylene bottles, stored on ice, and analysed within 24 h to minimise degradation.. Three independent hydroponic systems (control, *Chlorella vulgaris* and *Arthrospira platensis*) were sampled, with three reservoir sample per system per week, providing biological replication across treatments. Parameters that were measured for water analysis included dissolved oxygen (DO), turbidity, pH, electrical conductivity (EC), oxidation-reduction potential (ORP) and total dissolved solids (ppm) (Ng *et al*. 2021). Nutrient concentrations were determined using the Hach DR/2500 spectrophotometer (Hach 2001):


$$\begin{array}{l} \text{Orthophosphate }(\text{o-}{{\text{PO}}_{4}}^{3-}):\text{Ascorbic acid method (Method }8048)\\ \text{Nitrate-N }({{\text{NO}}_{3}}^{-}\text{-N}):\text{Cadmium reduction method (Method }8039)\\ \text{Ammoniacal-N }({\text{NH}}_{3}\text{-N}):\text{Salicylate method (Method }8155)\\ \text{Total nitrogen: Reactor digestion method (Method }8000) \end{array}$$

All instruments were calibrated prior to measurement, and blanks and standards were included in nutrient analyses to ensure accuracy. Analytical detection limits (DL) for each parameter were established based on instrument sensitivity and calibration standards. Values that fell below the detection limit were not reported as exact zeros; instead, they were denoted as “<DL” to indicate concentrations below the measurable threshold. This approach avoids misinterpretation of nutrient concentrations and ensures that reported values accurately reflect the limitations of the analytical methods.

### Statistical Analysis

Data were analysed using IBM SPSS Statistics Version 26 and STATISTICA 8. Plant growth parameters (height, weight, leaf number and leaf length) were measured on 10 plants per treatment, providing 10 biological replicates. For photosynthetic performance (Fv/Fm, rETRmax, α and Ek), pigment content (chlorophyll-a, carotenoids) and hydroponic water quality parameters (pH, EC, dissolved oxygen, turbidity, ORP, COD and nutrient concentrations), three replicates per treatment were analysed. Data were analysed using IBM SPSS Statistics Version 26 and STATISTICA 8. Two-way ANOVA was used to assess the effects of treatment and time on plant growth parameters (height, weight, fresh weight, leaf number and leaf length). Post hoc comparisons were performed using Tukey’s HSD test. A significance threshold of *p* < 0.05 was applied for all tests.

## RESULTS

### Plant Growth Studies

The growth of the Pak Choy over a period of 4 weeks of planting in hydroponic system was recorded. Fig. 2 and Table 1 show the visible growth of Pak Choy from week 1 to week 4 in three different treatments which are control, hydroponics treated with *Chlorella vulgaris* and treatment with *Arthrospira plantesis*. Based on the plant growth results, there is an significant increase (p < 0.05) in growth in the plant from week 1 to week 4. In week 4, the control group exhibited the highest fresh weight, measuring 9.344 g greater than the treatment group supplemented with *Chlorella vulgaris* inoculum, which recorded the second-highest fresh weight. Conversely, the *Arthrospira platensis* treatment demonstrated a significant increase (*p* < 0.05) over time but resulted in the lowest fresh weight overall. As for the height of the plants, the control group had the tallest plants by week 4 (106.651 cm), followed by those supplemented with *Chlorella vulgaris* and *Arthrospira platensis*.

### Photosynthetic Performance

Table 2 summarises the statistical comparison of key photosynthetic performance parameters such as Fv/Fm, α (alpha), rETR_max_ and Ek for Pak Choy grown under three treatment conditions (control, *Chlorella vulgaris* and *Arthrospira platensis*) over a four–week experimental period.

Over time, microalgae gradually colonised the reservoirs, influencing the photosynthetic performance of the plants. Across all treatments, Fv/Fm values remained relatively stable (ranged from 0.657 to 0.793), suggesting no significant differences in the maximum quantum yield of photosystem II. However, *Arthrospira platensis*-treated plants showed a decrease in Fv/Fm in week 4, though this change was not statistically significant (*p* > 0.05). In contrast, *Chlorella vulgaris*-treated plants demonstrated consistent improvement in photosynthetic efficiency. The rETR_max_ values increased in all treatments, indicating enhanced electron transport and photosynthetic capacity. This improvement was most notable in the control and *Chlorella vulgaris* groups, with *Chlorella vulgaris* showing a 21.8% increase in rETR_max_ from week 2 to week 4. Regarding Ek, all treatments showed adaptation to higher light intensities over time. The control group exhibited the highest Ek value in week 4 (158.010 ± 41.855 μmol photons m^−2^ s^−1^), suggesting a strong acclimation to light conditions.

### Microalgae Growth Performance

#### Pigmentation of microalgae

Table 3 presents the statistical comparison of pigmentation changes in carotenoids, chlorophyll *a*, phycocyanin and allophycocyanin in microalgae reservoirs subjected to different treatments. Pigment extraction was performed weekly over a four-week period for all sample groups: control, *Chlorella vulgaris* and *Arthrospira platensis*. The results indicate that *Chlorella vulgaris* exhibited significant increase (*p* < 0.05) in carotenoid content over time, peaking at week 4 with a difference of 1.511 mg/L compared to earlier weeks. *Arthrospira platensis* showed an increase in carotenoid levels at week 3, followed by a notable decline of 0.905 mg/L in week 4. The control group consistently displayed the lowest pigmentation levels across all four pigment types.

#### Photosynthetic performance of microalgae

Table 4 presents the statistical comparison of photosynthetic efficiency in water reservoirs subjected to different microalgae treatments: control, *Chlorella vulgaris and Arthrospira platensis*. Over time, microalgae gradually colonised the reservoirs, leading to measurable photosynthetic activity in subsequent weeks. All treatments exhibited comparable Fv/Fm values from week 2 onward, suggesting no significant differences in maximal photosystem II efficiency. However, *Arthrospira platensis* showed the highest α value in week 2 (1.52), which gradually declined to 0.938 by week 4, indicating a reduction in photosynthetic efficiency over time. *Chlorella vulgaris* demonstrated anincrease in photosynthetic performance, with consistently high values for α, rETR_max_ and Ek, particularly in weeks 3 and 4.

### Water Quality Analysis of the Reservoir

#### Water parameter assessments

Table 5 presents the statistical comparison of water quality parameters in reservoirs subjected to different microalgae treatments: control, *Chlorella vulgaris* and *Arthrospira platensis*. Weekly measurements were conducted over a four-week period to monitor changes in turbidity, electrical conductivity (EC), total dissolved solids (ppm), pH, oxidation-reduction potential (ORP) and dissolved oxygen (DO). Among the treatments, *Arthrospira platensis* exhibited the highest initial turbidity (24.800 ± 4.596 NTU in week 1), attributed to its larger biomass. This value dropped sharply to 3.093 ± 0.791 NTU by week 2 and further declined to 0.010 ± 0.000 NTU in week 3, indicating rapid sedimentation or biomass reduction. *Chlorella vulgaris* showed a similar trend, with turbidity peaking at 13.750 ± 0.594 NTU in week 3 before dropping drastically to 0.010 ± 0.000 NTU in week 4. In week 1, elevated turbidity was observed in the control reservoir containing synthetic fertiliser. This was likely due to the initial preparation of the nutrient solution, where incomplete dissolution and precipitation of fertiliser salts produced suspended particles. Physical disturbance from aeration and pumping may also have resuspended fine particulates, contributing to the cloudiness. The control group experienced a steady decline in turbidity, with a 90.7% reduction over four weeks. Electrical conductivity followed a consistent downward trend across all treatments, reflecting reduced ionic concentrations likely due to nutrient uptake by microalgae. *Arthrospira platensis* recorded the highest EC in week 1 (2.633 ± 0.062 mS/cm), which decreased to 1.683 ± 0.031 mS/cm by week 4. Similar patterns were observed in *Chlorella vulgaris* and the control group. Other parameters such as pH remained relatively stable across treatments, ranging between 7.45 and 7.57. ORP values decreased over time, particularly in the control group, suggesting changes in redox conditions. Dissolved oxygen levels varied, with *Arthrospira platensis* showing the highest DO in week 2 (8.733 ± 0.386 ppm), potentially indicating enhanced photosynthetic activity.

#### Nutrients concentration analysis

Table 6 presents the statistical comparison of nutrient concentrations in water reservoirs containing different microalgae treatments: control, *Chlorella vulgaris*, and *Arthrospira platensis*. Weekly measurements were taken over a four-week period to assess the concentrations of key nutrients, including orthophosphate (O-PO_4_^3−^), nitrate nitrogen (NO_3_^−^N), ammoniacal nitrogen (NH_3_-N) and chemical oxygen demand (COD). By week 4, all treatments showed a reduction in orthophosphate levels, with *Chlorella vulgaris* demonstrating the most effective phosphate absorption. *Chlorella vulgaris* also exhibited the highest efficiency in nitrogen uptake, followed closely by *Arthrospira platensis*, which retained higher levels of nitrate nitrogen. Ammoniacal nitrogen levels declined across all treatments, with *Arthrospira platensis* initially showing the highest concentrations.

In terms of COD reduction, both *Chlorella vulgaris* and *Arthrospira platensis* were effective, with *Chlorella vulgaris* showing a decrease from 276.667 ± 26.247 mg/L in week 1 to 56.000 ± 1.633 mg/L by week 4. Similarly, *Arthrospira platensis* reduced COD from 260.000 ± 8.287 mg/L to 22.333 ± 2.494 mg/L over the same period. The control group also showed a significant reduction, though less pronounced.

## DISCUSSION

### Plant Growth Analysis of Pak Choy

The results were analyzed to evaluate the effects of *A. platensis* and *C.vulgaris* on the growth rate and photosynthetic performance of Pak Choy cultivated in hydroponic systems. All of the plants has shown a general increase in their height, weight and leaf size as well. By week 4, which was marked as the final stage of the experiment, all treatments exhibited growth across multiple plant parameters. Among the treatments, the control group exhibited the greatest plant height; however, height alone does not fully represent overall plant performance, as the microalgae treatments influenced other growth parameters. Among the microalgae treatments, *C. vulgaris* demonstrated the most pronounced enhancement in plant growth. This can be attributed to its rich composition of phytohormones such as auxins, cytokinins and gibberellins, which are known to stimulate cell division, elongation and differentiation (Abreu *et al*. 2023). Consequently, *C. vulgaris*-treated plants exhibited superior root and shoot development compared to those treated with *A. platensis*. In a study that used microalgae as a fertiliser and was added to hydroponic systems, microalgae and cyanobacteria both demonstrated improve plant growth and morphological characteristics. For instance, as compared to controls, microalgal biomass or extracts from *Chlorella* sp. and *Anabaena* sp. considerably improved the stem length of cucumber seedlings used in that experiment by 81.7% and 58.3%, respectively. They simultaneously encouraged the development of lateral and fibrous roots at the expense of primary root elongation, suggesting enhanced root architecture associated with nutrient intake and vitality (Zhang *et al*. 2024). These variations in biomass distribution and morphological characteristics show how microalgal-derived biostimulants may enhance important hydroponic crop growth parameters.

Fig. 4 illustrates the distinct visual differences in roots observed in week 4 between Pak Choy plants treated with *C. vulgaris* and *A. platensis*. Notable variations were recorded in both root characteristics. Plants treated with *C. vulgaris* developed more elongated root systems, suggesting enhanced root extension and possibly improved nutrient uptake. In contrast, *A. platensis*-treated plants exhibited a more fibrous root structure accompanied by a darker green leaf coloration. These root characteristics differences may reflect species-specific effects of microalgae on root architecture and pigment accumulation, which could influence overall plant health and growth dynamics. Under controlled conditions, Pak Choy exhibited the most robust growth performance, indicating optimal development in the absence of any treatment. However, systems incorporating microalgae treatments specifically *C. vulgaris* and *A. platensis* demonstrated the capacity to support plant growth without the addition of synthetic fertilisers. Among these, *C. vulgaris* functioned effectively as a biostimulant, significantly enhancing leaf production and moderately improving other morphological parameters. In contrast, *A. platensis* showed limited efficacy, with modest gains in leaf development but comparatively weaker performance across other growth metrics. The green coloration is likely due to the absorption of chlorophyll derivatives and other pigment molecules released during *A. platensis* metabolism (Tan *et al*. 2017).

Root development is known to be influenced by auxins and cytokinins, two phytohormone-like compounds generated by microalgae such as *A. platensis* (Spirulina). Auxins, particularly indole-3-acetic acid (IAA), regulate root branching by encouraging lateral root initiation and boosting root hair density. Shorter primary roots and more extensive secondary roots may result from the phytohormone effect. The accumulation of chlorophyll in root tissues may be impacted by microalgal extracellular polysaccharides, which may improve nutrient absorption at the root surface and cause morphological and colour changes (Renganathan *et al*. 2024).

Interestingly, *A. platensis*-treated hydroponic systems produced a significant higher (p < 0.05) leaf count than *Chlorella vulgaris*, as recorded in Table 1 and Fig. 3. This may be due to its high content of amino acids, proteins, and growth factors, which promote cellular differentiation and early leaf initiation (Al-Badwy *et al*. 2023; Shedeed *et al*. 2022). Supporting this, Siringi *et al*. (2022) reported that *A. platensis*-treated lettuce (*Lactuca sativa*) developed more leaves and branches than untreated controls. However, increased leaf number does not necessarily correlate with overall plant size or biomass. The presence of antioxidants in *A. platensis* may contribute to healthier development and oxidative stress mitigation, potentially influencing leaf proliferation (Varia *et al*. 2022).

Additionally, the carotenoid content was significantly higher (*p* < 0.05) in *C. vulgaris*-treated reservoirs, which may have contributed to its growth performance. Carotenoids play a crucial role in photoprotection and light harvesting, enhancing plant vitality under varying light conditions. Microalgae growth typically follows four distinct phases: lag, log (exponential), stationary and death. Based on findings reported in our previous publication, the life cycle of microalgal cells is approximately 12 days (Tay *et al*. 2024; Ng *et al*. 2021). Therefore, measurements were taken at 4-day intervals to capture the physiological changes across these growth phases Microalgal treatments in treated plants have also been associated with increased levels of carotenoids and other photosynthetic pigments. Carotenoids like as β-carotene are vital for photoprotection and light harvesting, but they also serve as precursors to signalling molecules like abscisic acid (ABA), which can influence stress responses and metabolic pathways (Colarte *et al*. 2025). Studies show that using *C. vulgaris* to lettuce seedlings increases their levels of carotenoid and chlorophyll. This implies that more pigment molecules are being produced by the plants, most likely as a result of enhanced nutrient uptake, elevated metabolic activity, and hormonal signaling from algae biostimulants (Colarte *et al*. 2025).

The photosynthetic performance of Pak Choy remained consistent across all treatments, with no statistically significant differences observed, as shown in Table 2. This indicates that all plants maintained healthy photosynthetic activity throughout the four-week cultivation period, regardless of the microalgae treatment applied.

#### Microalgae Analysis

To evaluate the pigmentation and photosynthetic performance of *A.platensis* and *C. vulgaris*, both microalgae were monitored throughout the experiment. While *C. vulgaris* showed a substantial and statistically significant increase (P < 0.05) in carotenoid production, *A. platensis* exhibited comparatively lower pigment accumulation under the same conditions. The higher carotenoid content observed in *C. vulgaris* corresponds with its enhanced photosynthetic efficiency, as presented in Table 2. Given that carotenoids contribute to photoprotection and improved light utilisation(Sarsekeyeva *et al*. 2024), these findings are consistent with the more pronounced improvements in plant development observed in systems treated with *C. vulgaris*, while *A. platensis* still contributed measurable but less pronounced effects.

Both microalgae treatments showed an increase in Fv/Fm values, indicating enhanced photosynthetic efficiency and supporting the overall objectives of the study. However, while *A. platensis*—a cyanobacterium—typically produces higher levels of phycocyanin (PC) and allophycocyanin (APC), their synthesis may have been limited in this experiment due to environmental factors or interactions within the hydroponic system.

To enhance clarity, the purpose of each experimental assessment is explicitly outlined as follows:

1. Pigment analysis was conducted to quantify carotenoid and chlorophyll levels under each treatment.
2. Photosynthetic performance measurements were performed to evaluate lightuse efficiency.
3. Nutrient profiling was undertaken to examine how nitrogen and phosphorus availability influenced microalgal activity and plant responses.

Environmental constraints, particularly nutrient availability, appeared to influence pigment production. *A. platensis* exhibited lower concentrations of ammoniacal nitrogen (NH_4_^+^–N) and orthophosphate (OPO_4_^3−^) compared to *C. vulgaris* (Table 6). These limitations may have contributed to a slower transition of *A. platensis* from the lag phase into exponential growth, a response commonly observed when algal cultures acclimate to new environmental conditions (Thangavel *et al*. 2018). By week 4, orthophosphate levels had declined to zero across all treatments, indicating complete uptake by the plants. This nutrient depletion corresponded with increased plant growth, suggesting efficient nutrient utilisation and assimilation within the system.

### Water Quality Analysis of the Reservoir

The water quality assessment, which included dissolved oxygen (DO), electrical conductivity (EC), turbidity, pH, oxidation–reduction potential (ORP) and chemical oxygen demand (COD), was conducted to characterise the dynamic interactions between microalgae and the hydroponic environment. These parameters provide insight into how each microalgal strain modifies its surroundings and, consequently, its potential as a biostimulant. As shown in Table 5, turbidity peaked in week 3 for *C. vulgaris*, likely due to increased biomass accumulation, but subsequently declined by week 4, suggesting active nutrient assimilation and particle settling. In contrast, turbidity and DO trends in *A. platensis* were less favourable, indicating weaker contributions to water clarification and oxygenation. DO levels are a critical indicator of microalgal and root interactions, as higher DO enhances root respiration and nutrient uptake efficiency (Chowdury, *et al*. 2020). The stable pH observed across all treatments provided suitable conditions for both microalgal proliferation and plant nutrient availability.

Comparative evaluation of the two strains revealed that *A. platensis* demonstrated lower concentrations of ammoniacal nitrogen (NH_4_^+^–N) and orthophosphate (OPO_4_^3−^) than *C. vulgaris* (Table 6). This nutrient limitation may have delayed *A. platensis*’ transition from lag phase to exponential growth, an adaptation pattern commonly reported when cyanobacteria are introduced into new environments (Thangavel *et al*. 2018). Past studies similarly highlight that *Chlorella* spp. outperform *Arthrospira* in hydroponic systems due to faster growth rates, greater nitrogen and phosphorus assimilation, and stronger oxygenation capacity (Huo *et al*. 2020). These findings support the present observations, where *C. vulgaris* more effectively enhanced water quality and plant performance, although *A. platensis* still contributed measurable, albeit weaker, biostimulant effects.

The reduction in COD across all treatments, most notably in *C. vulgaris*, further indicates improved water quality. Lower COD reflects reduced organic loading, which minimises microbial oxygen consumption and enhances root-zone aeration (Aguilar-Torrejón *et al*. 2023). By week 4, orthophosphate levels declined to zero for all treatments, demonstrating complete nutrient uptake by plants. This nutrient depletion coincided with increased plant biomass accumulation, suggesting efficient nutrient utilisation within the microalgae hydroponic system. A limitation of this study is the relatively small sample size (*n* = 3) for several parameters. This constraint was due to space and resource availability, and results should therefore be interpreted with caution. Future studies with larger replication will be necessary to validate and extend these findings.

Beyond biological performance, costefficiency is an essential consideration for the adoption of microalgae-based biostimulants. *C. vulgaris* may present a more cost-effective option due to its rapid growth, high pigment productivity, and resilience under variable nutrient conditions, which reduce culture maintenance costs. In contrast, *A. platensis* often requires more controlled environmental conditions and may yield lower biomass within hydroponics, increasing operational costs. Future optimisation such as co-culturing both strains, integrating low-cost nutrient sources, or recycling microalgal biomass, could enhance overall system feasibility. Further research should evaluate long-term economic viability, scalability and the potential integration of microalgae into commercial hydroponic production frameworks.

## CONCLUSION

Overall, both microalgae treatments contributed positively to plant development, with increases observed in plant height and pigmentation. However, *C. vulgaris* consistently outperformed *A. platensis* across most growth parameters, including shoot and root development, likely due to its rich composition of phytohormones such as auxins, cytokinins and gibberellins. These hormones are known to promote cell division and elongation, enhancing overall plant vigor. While *A. platensis* exhibited a slower growth rate in terms of physical parameters, it increased the number of leaves produced throughout the experiment. This suggests that *A. platensis* may be particularly effective in promoting leaf initiation, possibly due to its high content of amino acids, proteins, and bioactive compounds that support cellular differentiation.

Nutrient analysis revealed a decrease in orthophosphate (O-PO_4_^3−^), ammoniacal nitrogen (NO_3_^−^-N) and chemical oxygen demand (COD) across all treatments by week 4, indicating effective nutrient uptake by the plants. Nitrate nitrogen (NO_3_^−^N) levels fluctuated throughout the experiment, suggesting variable assimilation rates. These findings reinforce the potential of *C. vulgaris*as a more efficient biostimulant in hydroponic systems. It is important to note that these results are specific to Pak Choy and may vary with different plant species.

The more rapid and consistent nitrate assimilation observed in *Chlorella*-treated systems reinforces *C. vulgaris* as the more efficient biostimulant; enhanced nitrate uptake aligns with the strain’s faster growth rate, higher pigment production, and stronger metabolic activity. Efficient nitrate assimilation is a key indicator of biostimulant potential because it reflects both microalgal productivity and the capacity to improve nutrient availability to the plant roots. In contrast, slower and more variable nitrate utilisation in *A. platensis* systems is consistent with its slower growth dynamics and more limited influence on plant performance.

This study demonstrates that while both microalgae can enhance certain aspects of plant growth and nutrient dynamics, *C. vulgaris* shows greater overall potential as a biostimulant for Pak Choy hydroponic cultivation. This study highlights the importance of matching microalgal species with specific crop and system conditions to optimise performance. As responses may vary with different plant species or environmental settings, further studies: especially those incorporating economic analysis, environmental optimisation, and longterm system performance are recommended to validate scalability and practical applicability.

## Figures and Tables

**FIGURE 1:**
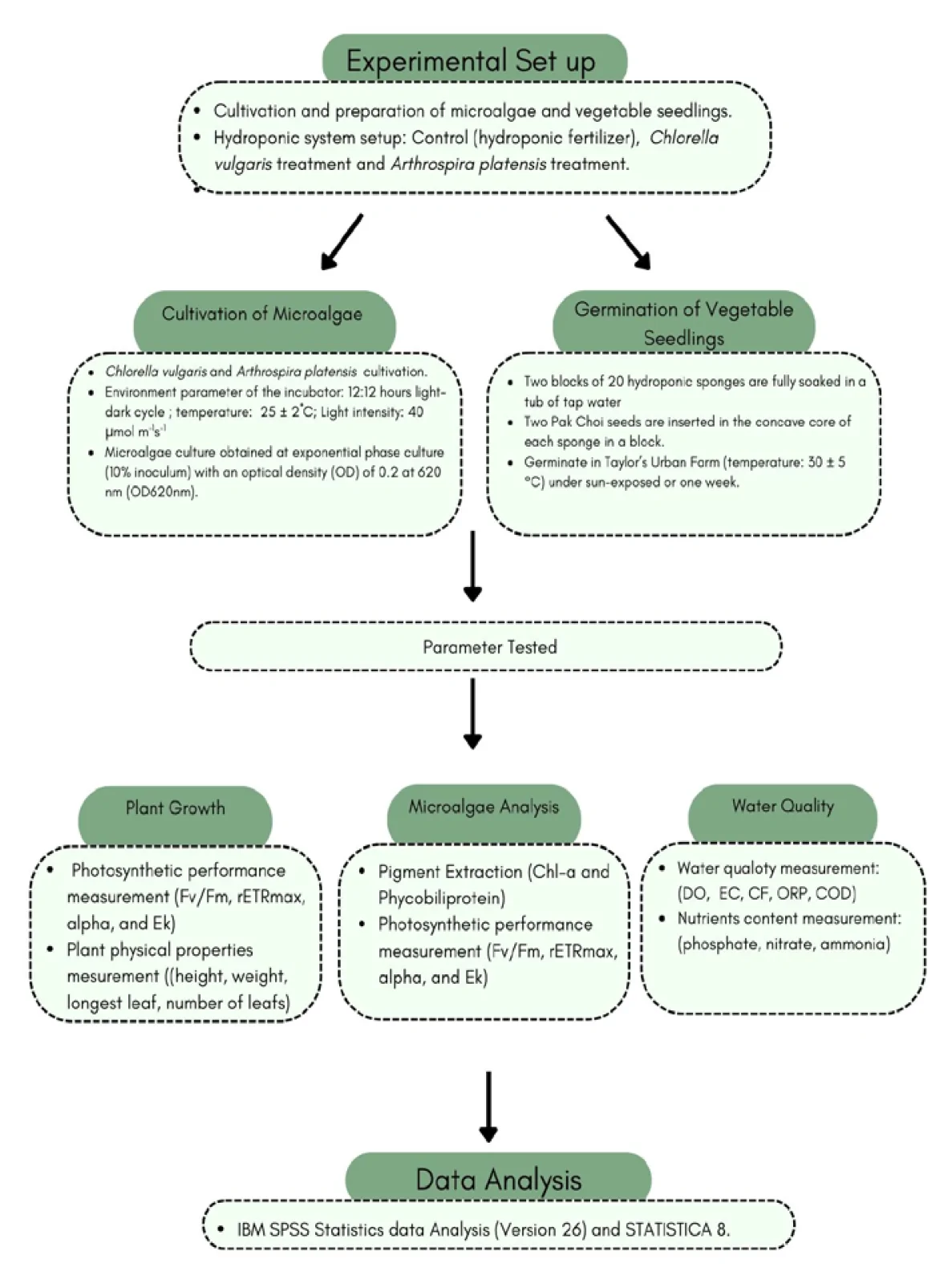
Experimental design.

**FIGURE 2:**
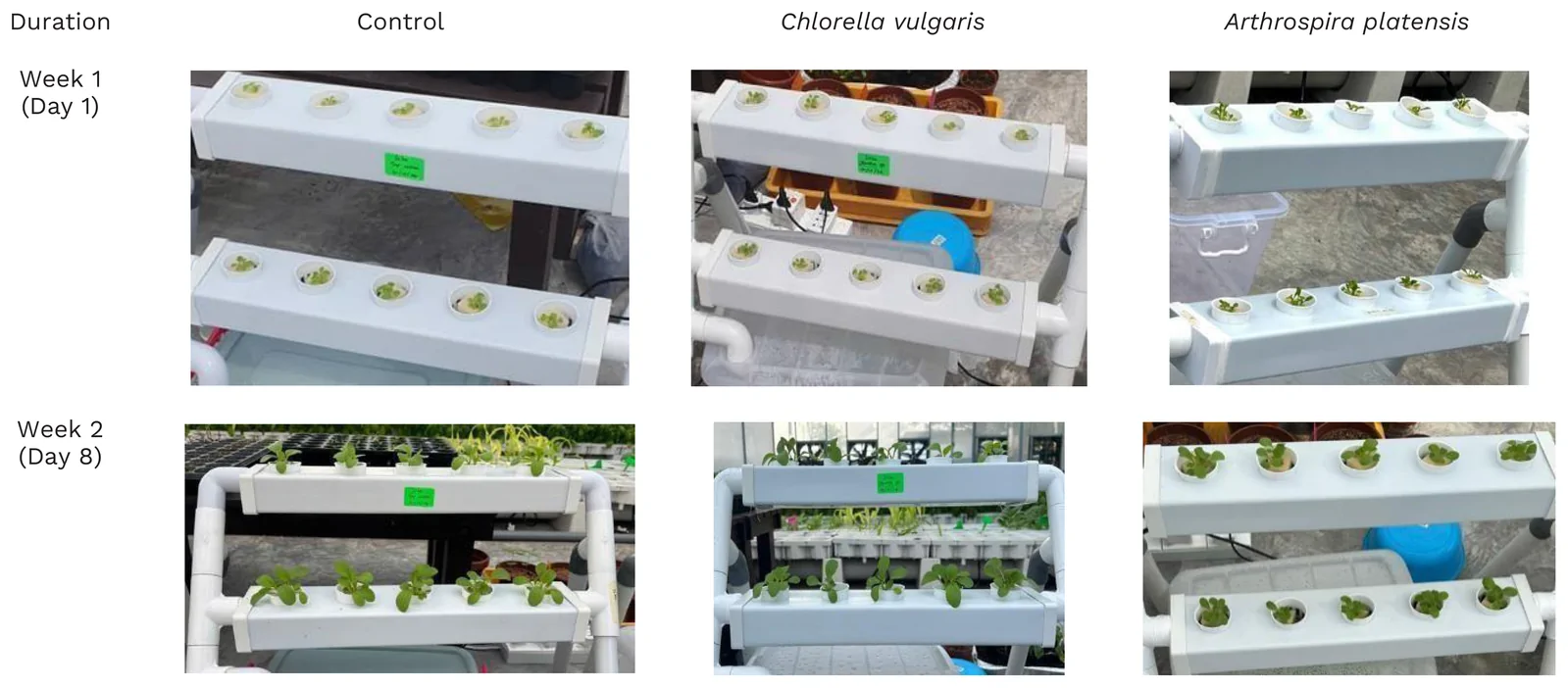

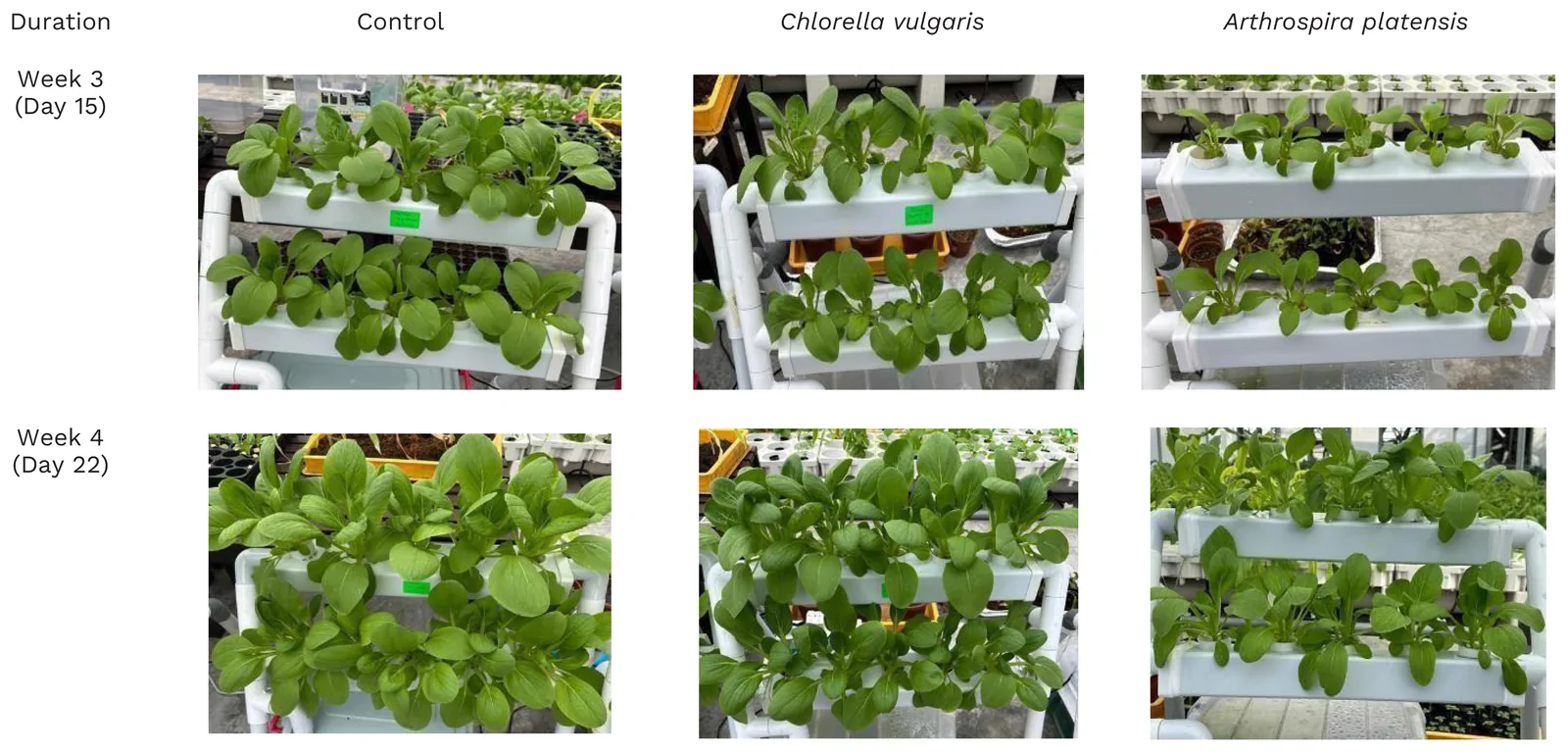
Growth of Pak Choy in hydroponic three hydroponic systems: control, *Chlorella vulgaris* treatment, and *Arthrospira platensis* treatment from week 1 to week 4.

**FIGURE 3:**
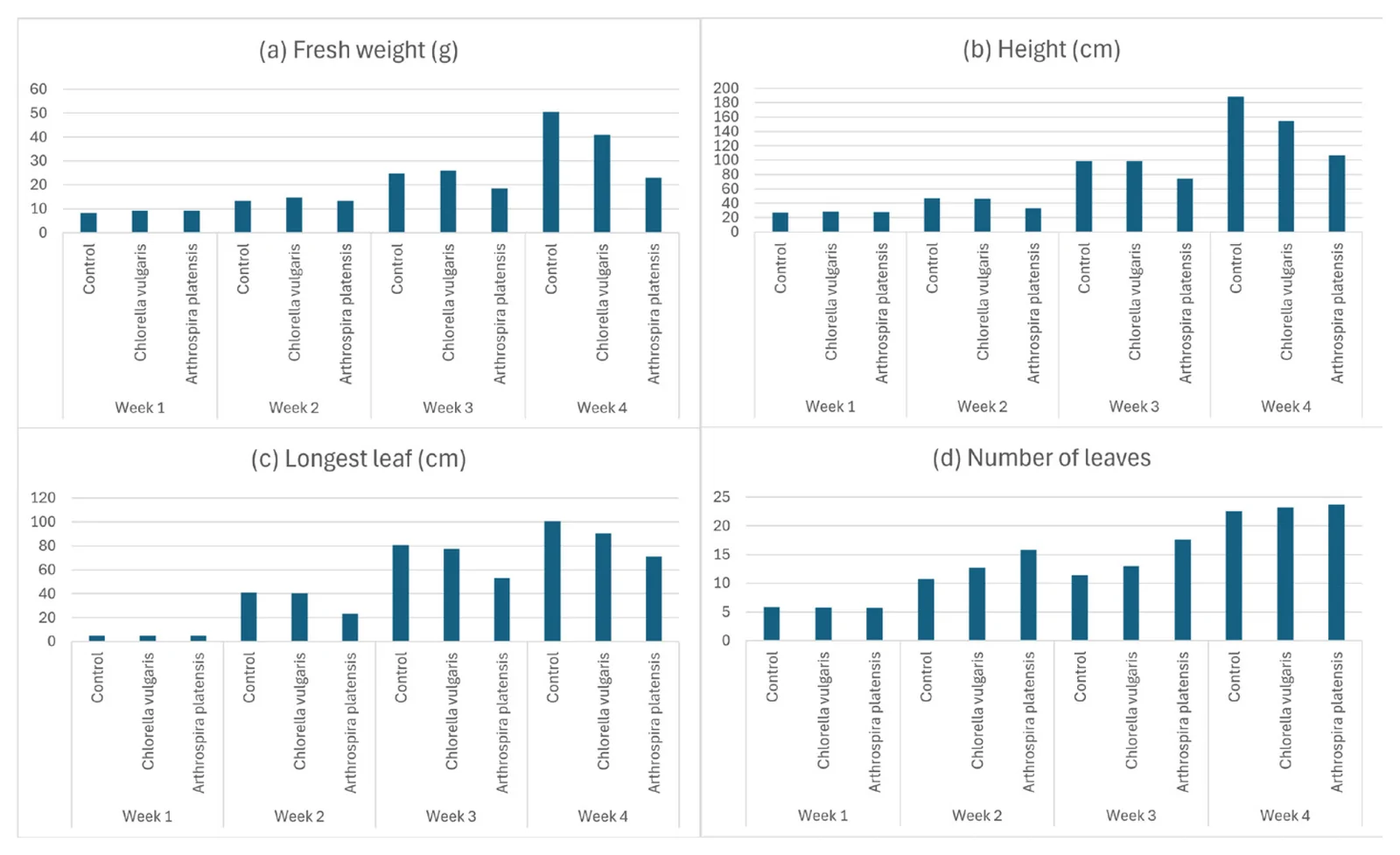
Comparison between the growth variables: (a) fresh weight, (b) height, (c) longest leaf and (d) number of leaves over 4 weeks.

**FIGURE 4:**
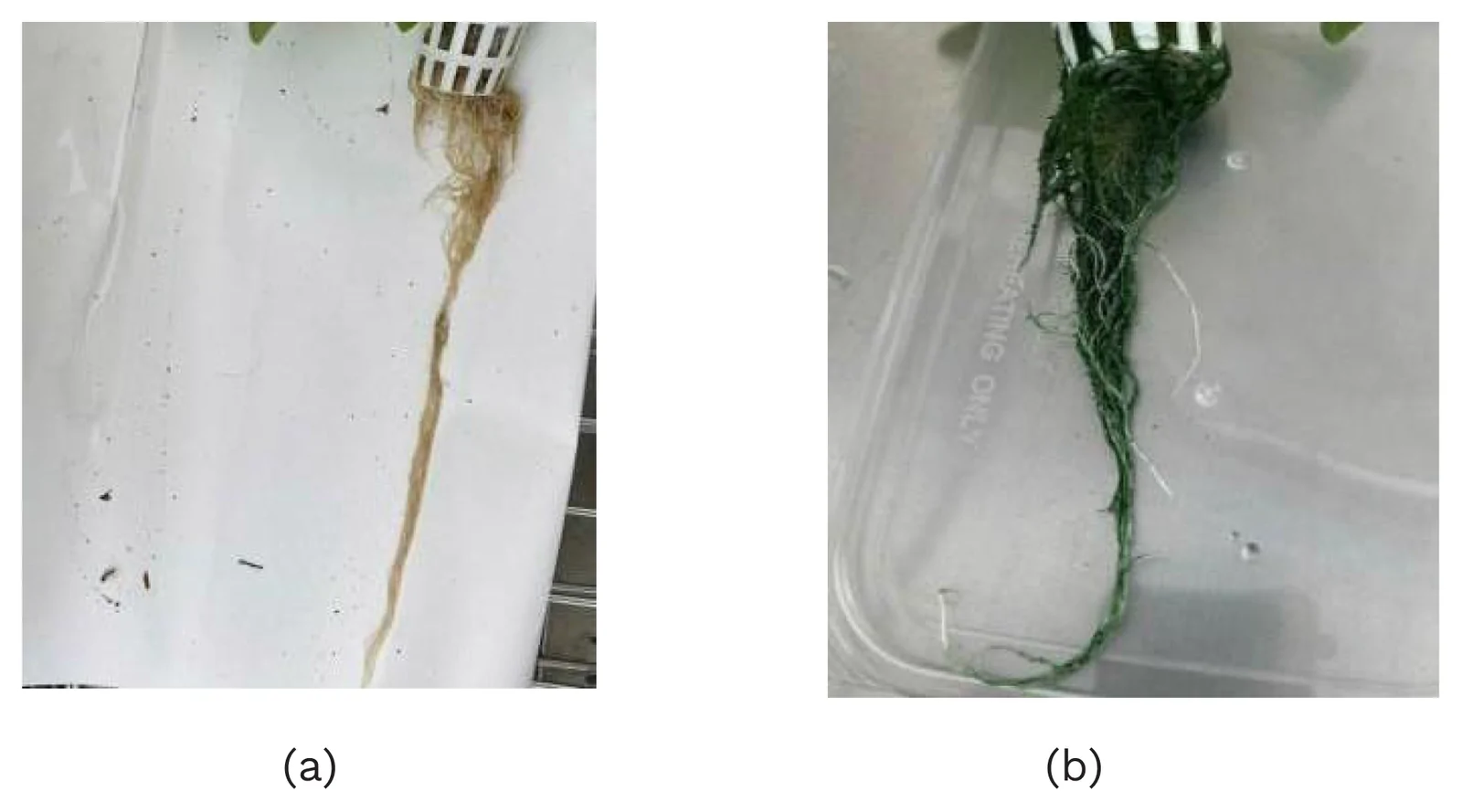
Root system morphology and leaf pigmentation of hydroponically grown plants at week 4 under (a) *Chlorella vulgaris* treatment and (b) *Arthrospira platensis* treatment.

**TABLE 1:** The statistical comparison of fresh weight, height, longest leaf and number of leaves in Pak Choy in different treatments; Control, *Chlorella vulgaris*, *Arthrospira platensis* throughout 4 weeks of growth.

Treatment	Duration	Fresh weight (g)	Height (cm)	Longest leaf (cm)	Number of leaves
Control	week 1	8.400 ± 3.040 ^f^	27.260±1.570 ^f^	5.000 **±** 0.000 ^h^	5.900 ± 0.943 ^e^
	week 2	13.353 ± 1.094 ^ef^	46.730 ± 6.808 ^e^	41.147 ± 4.331 ^f^	10.800 ± 1.887 ^d^
	week 3	24.810 ± 2.235 ^cd^	98.850 ± 7.834 c	80.61 ± 6.893 c	11.400 ± 1.497 cd
	week 4	50.435 ± 5.344 a	188.740 ± 13.517^a^	100.440 ± 5.586 a	22.600 ± 2.835 a
*Chlorella vulgaris*	week 1	9.200 ± 1.400 ^f^	28.150 ± 1.260 ^f^	5.000± 0.000 ^h^	5.800 ± 0.600 ^e^
	week 2	14.685 ± 2.702 ^ef^	46.390 ± 5.286 ^e^	40.360 ± 4.583 ^f^	12.700 ± 1.792 ^cd^
	week 3	25.977 ± 3.004 c	98.680 ± 7.973 c	77.520 ± 5.140 ^cd^	13.000 ± 1.844 ^cd^
	week 4	41.091 ± 7.617 b	154.410 ± 19.240 b	90.450 ± 3.488 b	23.200 ± 3.250 a
*Arthrospira platensis*	week 1	9.200 ± 1.077 ^f^	27.720 ± 1.685 ^f^	5.000±0.000 ^h^	5.700 ± 0.670 ^e^
	week 2	13.304 ± 2.627 ^ef^	33.130 ± 3.282 ^ef^	23.340 ± 3.078 ^g^	15.800 ±3.156 bc
	week 3	18.607 ± 6.883 ^de^	74.310 ± 11.539 ^d^	53.050 ± 6.058 ^e^	17.600 ± 3.072 b
	week 4	22.969 ± 4.337 ^cd^	106.650 ± 9.207 c	71.110 ± 6.341 ^d^	23.700 ± 6.482 a

*Notes*: Data were represented as means ± SD (*n* = 10). Different letters indicate significant differences between values. (ANOVA, Tukey’s HSD test, *p* < 0.05).

* abc indicates statistical differences.

Parameters with the same letter are not significantly different.

**TABLE 2:** The statistical comparison of Photosynthetic Performance Parameters (Fv/Fm, Alpha, rETRmax, and EK) of the Pak Choy in different treatments; control, *Chlorella vulgaris* and *Arthrospira platensis* throughout 4 weeks.

Treatment	Duration	Fv/Fm	Alpha (α)	rETR_max_ (μmol electrons m^−2^ s^−1^)	Ek (μmol photons m^−2^ s^−1^)
Control	week 2	0.710 ± 0.083 a	1.462 ± 0.397 ab	64.080 ± 6.28 a	47.531 ± 13.783 b
	week 3	0.657 ± 0.019 a	1.229 ± 0.134 abc	75.100 ± 14.817 a	61.296 ± 14.210 b
	week 4	0.737 ± 0.041 a	0.068 ± 0.086 c	105.010 ± 18.702 a	158.010 ± 41.855 a
*Chlorella vulgaris*	week 2	0.793 ± 0.017 a	1.424 ± 0.052 ab	67.386 ± 18.238 a	47.800 ± 14.579 b
	week 3	0.753 ± 0.029 a	1.168 ± 0.256 abc	72.682 ± 10.084 a	67.902 ± 26.237 b
	week 4	0.723 ± 0.038 a	0.682 ± 0.086 a	86.329 ± 31.221 a	55.336 ± 14.678 b
*Arthrospira platensis*	week 2	0.790 ± 0.016 a	1.525 ± 0.136 bc	91.909 ± 7.625 a	52.565 ± 3.589 ab
	week 3	0.683 ± 0.076 a	1.059 ± 0.155 abc	87.010 ± 17.599 a	86.011 ± 28.018 ab
	week 4	0.773 ± 0.012 a	0.938 ± 0.084 abc	83.376 ± 6.638 a	90.033 ± 13.588 ab

*Notes*: Data as means ± SD (*n* = 3). Different letters indicate significant difference between values (ANOVA, Tukey’s HSD test, *p* < 0.05).

* abc indicates statistical differences.

Parameters with the same letter are not significantly different.

**TABLE 3:** The statistical comparison of pigmentation changes in carotenoid, chlorophyll A, phycocyanin allophycocyanin of microalgae reservoir in different treatments; control, *Chlorella vulgaris* and *Arthrospira platensis reservoir. Data as means ± SD (n* = 3).

Treatment	Duration	Carotenoid (mg/L)	Chlorophyll a (mg/L)	PC (mg/L)	APC (mg/L)
Control	week 1	0.085 ± 0.097 b	0.915 ± 0.097 a	0.023 ± 0.002 a	0.019 ± 0.006 a
	week 2	0.075 ± 0.009 b	0.925 ± 0.012 a	0.023 ± 0.002^ab^	0.019 ± 0.006 a
	week 3	0.904 ± 0.065 ab	0.925 ± 0.009 a	0.001 ± 0.000 c	0.002 ± 0.001 a
	week 4	0.119 ± 0.049 b	0.896 ± 0.053 a	0.001 ± 0.000 c	0.001 ± 0.000 a
*Chlorella vulgaris*	week 1	0.048 ± 0.012 b	0.952 ± 0.012 a	0.008 ± 0.003 bc	0.010 ± 0.005 a
	week 2	0.760 ±0.030 ab	0.240 ± 0.030 a	0.008 ± 0.003^bc^	0.010 ± 0.005 a
	week 3	0.562 ± 0.116 ab	0.240 ± 0.030 a	0.001 ± 0.000 c	0.002 ± 0.000 a
	week 4	1.559 ± 1.236 a	0.973 ± 0.944 a	0.001 ± 0.000 c	0.002 ± 0.001 a
*Arthrospira platensis*	week 1	0.126 ± 0.041 b	0.874 ± 0.041 a	0.005 ± 0.007^bc^	0.007 ± 0.009 a
	week 2	0.173 ± 0.057 b	0.827 ± 0.057 a	0.005 ± 0.007 c	0.007 ± 0.009 a
	week 3	0.971 ± 0.015 ab	0.827 ± 0.057 a	0.000 ± 0.000 c	0.001 ± 0.000 a
	week 4	0.066 ± 0.026 b	0.934 ± 0.026 a	0.002 ± 0.002 c	0.006 ± 0.008 a

*Notes*:

* abc indicates statistical differences.

Parameters with the same letter are not significantly different.

**TABLE 4:** The statistical comparison of photosynthetic efficiency of the microalgae presented in water reservoir in different treatments; control, *Chlorella vulgaris* and *Arthrospira platensis*.

Treatment	Duration	Fv/Fm	Aplha	rETR_max_ (μmol electrons m^−2^ s^−1^)	Ek (μmol photons m^−2^ s^−1^)
Control	Week 1	0.000 ± 0.000 ^a^	0.000 ± 0.000 b	0.000 ± 0.000 ^a^	0.000 ± 0.000 ^a^
	Week 2	0.100 ± 0.070 ^bcd^	0.347 ± 0.220 ^ab^	45.195 ± 2.411 ^a^	187.016 ± 91.177 ^a^
	Week 3	0.090 ± 0.065 ^cd^	0.378 ± 0.206 ^ab^	95.025 ± 68.306 ^a^	467.775 ± 501.473 ^a^
	Week 4	0.070 ± 0.050 ^cd^	0.204 ± 0.095 ^ab^	21.314 ± 21.195 ^a^	69.269 ± 68.745 ^a^
*Chlorella vulgaris*	Week 1	0.573 ± 0.073 ^a^	0.487 ± 0.101 ^ab^	104.032 ± 22.484 ^a^	229.418 ± 88.376 ^a^
	Week 2	0.467 ± 0.281 ^ab^	0.568 ± 0.319 ^a^	65.230 ± 80.336 ^a^	153.524 ± 167.794 ^a^
	Week 3	0.740 ± 0.008 ^a^	0.635 ± 0.024 ^a^	188.141 ± 34.525 ^a^	298.372 ± 63.395 ^a^
	Week 4	0.727 ± 0.005 ^a^	0.661 ± 0.151 ^a^	124.462 ± 22.428 ^a^	203.956 ± 70.893 ^a^
*Arthrospira platensis*	Week 1	0.080 ± 0.113 ^cd^	0.166 ± 0.123 ^ab^	59.486 ± 13.683 ^a^	260.690 ± 106.988 ^a^
	Week 2	0.660 ± 0.037 ^a^	0.382 ± 0.128 ^ab^	153.612 ± 60.965 ^a^	503.227 ± 358.832 ^a^
	Week 3	0.417 ± 0.085 ^abc^	0.515 ± 0.108 ^ab^	88.598 ± 14.708 ^a^	385.398 ± 358.008 ^a^
	Week 4	0.447 ± 0.019 ^abc^	0.622 ± 0.077 ^a^	88.598 ± 14.708 ^a^	144.546 ± 30.804 ^a^

*Notes*: Data as means ± SD (*n* = 3) with Anova, Tukey’s HSD test, *p* < 0.05.

* Means with ^abc^ indicate statistical differences. Parameters with the same letter are not significantly different.

**TABLE 5:** The statistical comparison of water quality parameters in the reservoir in different reservoirs of microalgae; control, *Chlorella vulgaris* and *Arthrospira platensis*.

Treatment	Duration (week)	Turbidity (NTU)	Electrical conductivity (mS/cm)	Total dissolved solids (ppm)	pH	ORP (mv)	Dissolved oxygen (ppm)
Control	1	7.89 ± 0.178 ac	1.943 ± 0.017 de	756.667 ± 3.091 abcd	7.500 ± 0.00 a	179.667 ± 6.182 cd	5.733 ± 0.435 c
	2	0.913 ± 0.062 e	2.090 ± 0.014 c	410.333 ± 249.032 dc	7.450 ± 0.033 a	193.000 ± 4.967 bcd	7.393 ± 0.619 abc
	3	0.670 ± 0.353 e	1.507 ± 0.017 g	705.333 ± 233.345 abcd	7.537 ± 0.005 a	255.333 ± 10.209 a	6.470 ± 0.148 bc
	4	0.730 ± 0.0850 e	0.650 ± 0.000 h	252.000 ± 61.530 d	7.577 ± 0.017 a	97.000 ± 2.828 f	6.047 ± 0.528 bc
*Chlorella vulgaris*	1	9.123 ± 0.167 cb	1.930 ± 0.022 e	562.667 ± 271.657 dcb	7.500 ± 0.000 a	206.000 ± 3.742 bc	6.053 ± 0.482 bc
	2	9.200 ± 0.062 cb	2.047 ± 0.014 cd	919.333 ± 54.652 abcd	7.460 ± 0.036 a	213.333 ±2.357 bc	6.787 ± 1.429 abc
	3	13.750 ± 0.594 b	1.660 ± 0.024 f	678.000 ± 116.793 abcd	7.537 ± 0.009 a	224.333 ± 9.286 ab	8.017 ± 0.494 ab
	4	0.010 ± 0.000 e	0.240 ± 0.005 i	104.333 ± 0.943 e	7.557 ± 0.017 a	112.667 ± 24.445 f	7.403 ± 0.608 abc
*Arthrospira platensis*	1	24.800 ± 4.596 a	2.633 ± 0.062 a	1156.667 ± 9.428 a	7.453 ± 0.009 a	160.333 ± 8.340 de	7.257 ± 0.258 abc
	2	3.093 ± 0.791 ae	2.443 ± 0.059 b	985.000 ± 233.345 ab	7.570 ± 0.014 a	160.333 ± 8340 de	8.733 ± 0.386 a
	3	0.010 ± 0.000 e	0.650 ± 0.000 cd	559.667 ± 290. 425 dcb	7.570 ± 0.014 a	128.000 ± 13.491 ef	8.030 ± 0.096 ab
	4	3.337 ± 0.580 ae	1.683 ± 0.031 f	795.000 ± 13.367 abcd	7.537 ± 0.021 a	119.333 ± 0.471 f	7.410 ± 0.893 abc

*Notes*: Data as means ± SD (*n* = 3) with Anova, Tukey’s HSD test, *p* < 0.05.

* abc indicate statistical differences across weeks and treatments.

Parameters with the same letter are not significantly different.

**TABLE 6:** The statistical comparison of nutrient analysis of the water in the reservoir in different reservoirs of microalgae; control, *Chlorella vulgaris* and *Arthrospira platensis*.

Treatment	Duration	Orthophosphateo-PO_4_^3−^ (mg/L)	Nitrate nitrogen NO_3_^−^N (mg/L)	Ammoniacal nitrogen NH_3_-N (mg/L)	COD (mg/L)
Control	week 1	0.483 ± 0.026 cba	5.633 ± 1.167 ba	0.600 ± 0.008 a	251.333 ± 8.654^a^
	week 2	0.623 ± 0.009 b	4.867 ±0.759 ba	0.087 ± 0.005 a	97.667 ± 2.055 c
	week 3	0.260 ± 0.008 cbd	4.800 ±0.535 ba	0.037 ± 0.033 a	22.000 ± 2.160 gf
	week 4	<DL	0.900 ± 0.000 dc	0.037 ± 0.033 a	3.742 ± 0.047 f
*Chlorella vulgaris*	week 1	0.557 ± 0.005 ab	6.000 ± 0.245 ba	0.047 ± 0.005 a	276.667 ± 26.247 a
	week 2	0.697 ± 0.017 a	3.333 ± 0.903 bdc	0.110 ± 0.037 a	152.667 ± 8.994 b
	week 3	0.257 ± 0.243 dcb	5.633 ± 0.386 ba	0.037 ± 0.009 a	61.000 ± 2.944 de
	week 4	<DL	0.300 ± 0.000 d	<DL	56.000 ± 1.633 de
*Arthrospira platensis*	week 1	0.467 ± 0.165 cba	3.133 ± 2.155 bdc	1.393 ± 1.843 a	260.000 ± 8.287 a
	week 2	0.147 ± 0.172 dc	7.300 ± 1.105 a	0.067 ± 0.012 a	86.333 ± 1.247 cd
	week 3	0.043 ± 0.009 c	6.867 ± 0.411 bc	0.010 ± 0.000 a	48.667 ± 4.989 ef
	week 4	<DL	3.533 ± 0.419 a	<DL	22.333 ± 2.494 f

*Notes*: Data as means ± SD (*n* = 3) with Anova, Tukey’s HSD test *p* < 0.05.

* abc shows the contents with significantly difference across weeks and treatments.

Parameters with the same letter are not significantly different. < DL= below the detection limits.

## APPENDICES

FIGURE S1 External view of Taylor’s Urban Farm at Taylor’s University, Malaysia.

FIGURE S2 Layout of Taylor’s Urban Farm with the 3 elements of the cooling system. (1) - Cooling pad (green) and 6 exhaust fans (red). (2) - White Meiryo shading net. (3) - Double door entry.
